# Overview of Methotrexate Toxicity: A Comprehensive Literature Review

**DOI:** 10.7759/cureus.29518

**Published:** 2022-09-23

**Authors:** Khalid M Hamed, Ibrahim M Dighriri, Abdulrahman F Baomar, Baidaa T Alharthy, Foza E Alenazi, Gadheer H Alali, Rawan H Alenazy, Nidaa T Alhumaidi, Dania H Alhulayfi, Yasmen B Alotaibi, Sarah S Alhumaidan, Zahra A Alhaddad, Adhwa'a A Humadi, Shahad A Alzahrani, Rahaf H Alobaid

**Affiliations:** 1 Clinical Toxicology, Umm Al-Qura University, Taif, SAU; 2 Pharmacy, King Abdulaziz Specialist Hospital, Taif, SAU; 3 Pharmacy, Chronic Care Specialized Medical Hospital, Jeddah, SAU; 4 Pharmaceutical Care, General Network for Healthcare Providers Hospital, Jeddah, SAU; 5 Pharmacy, Al Aziziyah Children Hospital, Jeddah, SAU; 6 Pharmacy, Dallah Health Company, Khobar, SAU; 7 General Medicine and Surgery, Northern Border University, Arar, SAU; 8 Pharmacy, Taif University, Taif, SAU; 9 Pharmacy, Qassim University, Unaizah, SAU; 10 Pharmacy, King Faisal University, Al-Ahsa, SAU; 11 Pharmacy, Jazan University, Jazan, SAU; 12 Pharmacy, Umm Al-Qura University, Taif, SAU; 13 Pharmacy, Al-Dawaa Medical Services Company, Riyadh, SAU

**Keywords:** overdose, dmard, rheumatoid arthritis, hdmtx, toxicity, methotrexate

## Abstract

Methotrexate (MTX) is significantly more effective than and has a considerable advantage over placebo in patients with severe and persistent rheumatoid arthritis (RA). The drug is used to treat a variety of malignant disorders (leukemia and cancer of the lung, breast, and uterus) and ectopic pregnancy. As its side effects are outweighed by its effectiveness, MTX is a first-line antirheumatic drug in many countries. MTX is found in extracellular compartments, such as the synovium, as well as other organs, such as the kidney and liver. To improve treatment, increase adherence, and decrease mortality in MTX therapy, it is essential to reduce its toxicity and understand its side effects. Therefore, this comprehensive review was conducted to assist physicians and researchers in better understanding the toxicity of MTX and how to deal with this toxicity. MTX is eliminated via the kidneys, which are capable of excretion and reabsorption within the renal tubules. Although higher doses of MTX (known as high-dose MTX (HD-MTX), defined as doses of 500 mg/m^2^ or greater) are often more beneficial, they can produce toxicity and side effects such as bone marrow suppression, pulmonary toxicity, nephrotoxicity, hematologic toxicity, and an increased risk of infections. Treatment of severe MTX toxicity has three main goals: clearance of MTX from the bloodstream, folinic acid therapy, and organ treatment. Leucovorin is highly beneficial in preventing myelosuppression, gastric toxicity, and neurotoxic effects after HD-MTX therapy. The preferred antidote for MTX poisoning is folinic acid. Glucarpidase has been licensed for the treatment of high plasma MTX levels of >1 μmol/L in patients with compromised renal function who have delayed MTX elimination. In patients with renal deficiency, a lower initial dose is considered with an estimated glomerular filtration rate (eGFR) between 30 and 59 mL/minute. These patients need to be monitored, and a more gradual dosage increase and a lower weekly maximum should be considered regarding their general health situation. MTX is contraindicated in patients with RA if the eGFR is <30 mL/minute.

## Introduction and background

Folate inhibitors were among the earliest anticancer drugs produced. Aminopterin was first used to promote remission in children with acute lymphoblastic leukemia (ALL) in 1948, and the related drug methotrexate (MTX) is currently an essential component of contemporary ALL therapy [1]. MTX has been used to treat rheumatoid arthritis (RA) and psoriasis since 1951 [2]. However, MTX therapy for RA was not widely used until the 1980s. Later, it was shown to be significantly more effective than and to have a considerable advantage over placebo in patients with severe and persistent RA [3]. In addition, it is used today to treat a variety of malignant disorders and ectopic pregnancy [4,5]. In 1988, the Food and Drug Administration (FDA) approved MTX as an RA treatment [6]. MTX can be combined with other biological disease-modifying antirheumatic drugs (DMARDs) with potential efficiency improvements [7]. As its side effects are outweighed by its effectiveness, MTX is a first-line antirheumatic drug in many countries [8].

MTX may suppress the production of dihydrofolate reductase and decrease the stocks of tetrahydrofolate, both of which are required for the production of purine nucleotides and thymidylate, which are both required for cell replication and DNA synthesis. Cytotoxic MTX works mainly in rapidly multiplying cells, such as lymphocytes, which explains its significant anti-inflammatory, immunosuppressive, and apoptosis properties [8].

High-dose MTX (HD-MTX) is defined as doses of 500 mg/m^2^ or greater. Although higher doses are often more beneficial, they can produce toxicity and side effects such as bone marrow suppression, pulmonary toxicity, nephrotoxicity, hematological toxicity, and an increased risk of infections [9,10]. Previous research has found that, after taking HD-MTX, 60% of people have reversible hepatitis, and 25% develop hyperbilirubinemia [11]. The likelihood of nephrotoxicity in lymphoma patients receiving HD-MTX treatment is 9.1% [12]. MTX excretion delay is defined as MTX levels of >1 μmol/L at approximately 48 hours and then 0.1 μmol/L at 72 hours [13]. MTX should only be used to treat life-threatening neoplastic diseases or persistent severe RA or psoriasis that do not respond well to other treatments [14,15].

After a lethal dose of MTX, it will take two to three days, during which the patient will receive several leucovorin doses, for the harmful effects of MTX to end. Rapid clearance of MTX by the kidneys is needed for leucovorin to achieve optimal recovery, which requires extensive pretreatment as well as follow-up treatment fluids and urine alkalinization [16]. The main toxicity of HD-MTX includes elevated blood transaminase concentrations and renal impairment, which might cause delayed clearance of the medication [16]. As a result, reducing toxicity and understanding the side effects of HD-MTX can result in improved treatment, increased adherence, and decreased mortality. Therefore, this comprehensive review of the literature will assist physicians and researchers in better understanding the toxicity of MTX and how to deal with this toxicity.

## Review

Pharmacokinetics of MTX

When administered by the mouth, MTX is quickly but inefficiently absorbed, a process that exhibits interindividual heterogeneity but not as a function of concurrent meal consumption. The protein bound to albumin is around 50% [17]. MTX is found in extracellular compartments, such as the synovium, as well as other organs, such as the kidney and liver. Liver aldehyde oxidase might transform MTX to 7-hydroxymethotrexate, which can later be transformed to MTX polyglutamyl (MTXPG) derivatives by the enzyme folylpolyglutamyl synthase (FPGS) and preferentially maintained within cells. This is a continuous process in which glutamate residues are removed in the direction of gamma-glutamyl hydrolase (GGH). MTX is eliminated via the kidneys, which are capable of excretion and reabsorption within the renal tubules. Because these mechanisms saturate at various locations, non-linear excretion kinetics might occur [17]. Polyglutamation pharmacokinetics are known to vary in psoriatic and RA patients undergoing MTX treatment. This is considered an important step in the mode of action of MTX in inflamed circumstances because the higher-order MTXPG induces a persistent accumulation of adenosine, which subsequently mediates part of the anti-inflammatory effects of MTX [18]. The reason for polyglutamation diversity is genetic diversity between the two enzymes, folylpolyglutamyl synthase (FPGS) and gamma-glutamyl hydrolase (GGH), resulting in variable action in one or both of the enzymes [18].

MTX toxicity

MTX may be dangerous if administered improperly. The most serious possible adverse effect is severe myelosuppression, which causes the majority of the relatively infrequent fatalities caused by MTX [19]. Other side effects include bone marrow suppression, liver fibrosis, pneumonitis, homeopathy, and baldness [20,21]. Hepatitis, kidney dysfunction, hyperglycemia, and being overweight are all risk factors for MTX [22]. MTX is a hepatotoxic drug that may cause cirrhosis and hepatitis [23]; the initial technique of delivering MTX in modest daily doses, which is no longer in use, exacerbated this effect. Cirrhosis is very rare at cumulative doses of <1.5 g [23]. A significant number of patients discontinue MTX treatment due to intolerable toxicity; therefore, variables such as genetic differences, which may enable the prediction of such a result, could alleviate suffering and even save lives.

Liver Toxicity of MTX

A study from 1971 found altered liver functions in patients when using MTX to treat psoriasis [24]. A study from 1989 reported that hepatotoxicity in patients with RA could reach 70% during the first two to four years of MTX treatment [25]. Research in 2010 discovered that increased liver enzymes, notably alanine aminotransferase and aspartate aminotransferase, were associated with MTX [26]. The method by which MTX induces hepatotoxicity has not yet been determined; however, it is thought to be connected to the cellular pathways of the drug [27]. There are various possibilities, one of which is that MTX activates Ito cells in the liver [28]. When Ito cells are triggered by prolonged liver damage, they shift to myofibroblasts, which are producers of collagen and other matrix proteins, such as fibronectin, resulting in cell enlargement [29]. Another possibility is that long-term intracellular storage of MTX, particularly MTX metabolites (γ-polyglutamates), results in a chronic loss of folate, a component required for deoxyribonucleic acid (DNA) production [30]. Furthermore, while hepatotoxicity is uncommon, it can be exacerbated by certain risk factors, including a family history of hereditary liver failure, a history of alcohol intake, diabetes, a lack of folate supplementation, exposure to elevated doses of hepatotoxic chemical agents, and dyslipidemia [30,31].

Kidney Toxicity of MTX

A study published in 2003 found a link between MTX and kidney damage as well as the possibility of mortality in individuals with kidney failure [32]. The reason that MTX-induced renal dysfunction is a fundamental problem is that renal function in RA patients is already compromised. Because the renal tubules excrete more than 90% of MTX, any kidney problem may cause MTX to be removed poorly. Prolonged, persistent, or increased plasma levels of MTX are a consequence and may result in inefficient leucovorin rescue and a significant increase in MTX-related toxicities. The precipitation of MTX and its metabolites in the renal tubules was assumed to be the cause of renal impairment, although this is challenged by the finding that MTX may have a direct toxic influence on the kidney tubules. According to 2006 research, MTX causes renal failure because of MTX-induced kidney failure and renal tubular enlargement [33]. To predict the development of kidney failure, regular monitoring of plasma and serum creatinine MTX levels is essential as soon as MTX treatment begins. Recent research has shown that biomarkers such as kidney injury molecule-1 (KIM-1) and cystatin C can be used to diagnose kidney damage [34,35]. One symptomatic treatment to avoid MTX-related nephritis and the precipitation of MTX and its metabolites is the alkalinization of the urine [36].

Hematological Toxicity of MTX

MTX-treated RA patients can have hematological damage, including myelosuppression, leukopenia, neutropenia, and megaloblastic anemia [37]. Furthermore, hematological toxicities result in up to 25% of people discontinuing treatment due to the risk of mortality. Pancytopenia is among MTX toxicities and is difficult to prevent since it can appear unexpectedly during therapy [38]. Although the actual mechanism of MTX-induced hematological toxicity is unknown, it has been connected to the genesis of RA. One source of MTX-induced hematopoietic toxicity has been identified as excess unbound extracellular MTX. Another study found that MTX-related neutropenia may be influenced by the sociocultural status, cognitive capabilities, and distress of patients [39]. Furthermore, recent research found that the main cause of MTX-induced thrombocytopenia was that MTX increased platelet apoptosis, resulting in mitochondrial dysfunction [40]. Age, infections, folic acid deficiency, hypoalbuminemia, and concomitant medications are all issues to consider. In 2009, research reported that renal function impairment was a substantial risk factor for the development of hematological toxicity of MTX. MTX may increase the risk of pancytopenia-related death, especially in dialysis patients [41]. Another study revealed that approximately 3% to 4% of patients treated with MTX for RA have thrombocytopenia [42]. A 1998 study found considerable thrombocytopenia in people taking MTX with nonsteroidal anti-inflammatory drugs [43]. The occurrence of leukopenia can be detected after one to three weeks of MTX treatment [38]. According to the research findings, the immunosuppressive effects of MTX administration increase the frequency of infection, and the intensity of the infection illness also increases [44].

Pulmonary Toxicity of MTX

According to one study, more than 25% of patients who receive MTX treatment have coughing, wheezing, breathlessness, or other respiratory problems [45]. Research published in 2014 found that MTX therapy increases the risk of lung disease in patients compared to other DMARDs [46]. A 2009 study discovered that MTX causes lung damage due to cytokine release [47]. Individuals may have respiratory side effects after four weeks of starting MTX, which are likely to be caused by idiosyncratic immunological responses [48]. Fibrosis, interstitial pneumonitis, or even substantial alveolar destruction may be the result of MTX-induced respiratory toxicity [45]. Long-term MTX therapy was shown to cause alveolar epithelial cell damage and lung fibrosis in an animal model [49]. According to case-control studies, several risk factors have been proposed, including rheumatoid pleuropulmonary involvement, old age, diabetes, hypovolemia, and previous use of DMARDs [50,51]. These respiratory adverse effects might be associated with rheumatoid symptoms. As a consequence, RA patients receiving MTX should be continuously monitored in the event of respiratory symptoms or the possibility of lung injury [52].

MTX Increases Infection Risk

The research found that various stages and profiles of MTX treatment might contribute to varying infection risks in patients. As a result, RA patients are more vulnerable to increased infectious complications within the first year of MTX therapy. People with severe RA have also been shown to be more susceptible than those with moderate or mild RA [27]. According to a published article, patients with RA had infections as a result of the use of MTX [53]. Cryptococcosis, herpes zoster eruptions, and secondary infections are the most prevalent bacterial infections after MTX therapy [54]. There have also been cases of *Nocardia asteroides*, *Nocardia brasiliensis*, and *Pneumocystis carinii* pneumonia infections [55]. A 2014 case report found that some MTX-treated RA individuals were infected with pro-oncogenic viruses and developed intraoral ulcers, which eventually progressed to Hodgkin's lymphoma [56]. To limit the risk of infection, the use of MTX in conjunction with other traditional immunosuppressive DMARDs should be carefully examined. According to a 2007 study, patients should not receive vaccines other than those for influenza and pneumococcal disease [57].

Carcinogenicity of MTX

Case reports on MTX toxicity have linked MTX to several forms of malignancy, namely pseudolymphoma and lymphomas [58]. However, a research paper published in 1999 could not find a link between MTX treatment and proven oncogenicity due to a lack of solid data [59]. Malignancies have been associated with RA because the autoimmune pathogenesis of RA may play a role in cancer progression [60]. MTX has been linked with lymphoproliferative disorders [61]. A study found a link between MTX treatment and an increased risk of lymphoma [62]. Additional research has validated the conclusion of the previous studies that there are MTX-associated lymphoproliferative diseases [63].

Dermatological Toxicity of MTX

MTX is an anti-inflammatory medication used to cure psoriasis [64]. Its toxicity is rare given low doses, a proper dose schedule, and adherence to guidelines [65]. MTX may still induce dermatological toxicity. The dermatological adverse effects of MTX treatment range from minor to severe [66]. MTX toxicity may appear as bone marrow suppression and gastrointestinal ulcers. Other unusual but often observed characteristics include cutaneous ulceration within skin lesions in individuals with underlying psoriasis vulgaris [67,68]. MTX may cause minor to severe dermatological side effects [69]. MTX toxicity is enhanced by drugs that reduce renal elimination, including sulfonamides, aminoglycosides, cisplatin, penicillins, and colchicine, as well as by drugs that displace methotrexate from protein binding sites in plasma, including sulfonamides, phenytoin, retinoids, and barbiturates [70]. Before starting MTX treatment, a viable pregnancy must be avoided, and kidney and liver function tests and liver enzymes, as well as a complete blood count, must be performed. A complete blood count must be obtained seven days after starting MTX as well as after subsequent dosage increases. Blood counts should be checked every two to four weeks for the first few months [71]. MTX’s toxic effects must be treated early. Dermatologists must detect clinical and histopathological poisoning. The characteristic histology of cutaneous MTX toxicity, including keratinocyte enlargement and epidermal necrolysis, confirms clinical observations of direct toxic action [72]. Dermatological side effects, such as nonspecific morbilliform drug rashes, which are erythematous, macular, itchy, and limited to the neck and trunk, are reported to affect 14% to 15% of people. Under extreme circumstances, MTX may cause photoreactivation, photo intensification, and skin hyperpigmentation [73,74]. In dermatology, the initial doses of normal oral methotrexate vary from 5 to 15 mg per week, increasing after two to four weeks to a maximum dose of 25 mg per week [75]. A case series study determined the severe cumulative toxic dose and period of MTX in psoriasis; the research found that a severe cumulative dose of MTX, which ranged from 35 mg to 150 mg taken over three to seven days, was what caused toxic effects in patients [76].

Gastrointestinal Side Effects of MTX

The main dose-limiting issue for the use of MTX is gastrointestinal toxicity. For patients, MTX-induced intestinal mucositis is a serious burden. It can affect the entire digestive system and is often accompanied by nausea, stomach pain, and cramping. These symptoms often lead to malabsorption, weight loss, and interruption of medications [77]. Previous research in 1989 found that 20% to 70% of patients with RA reported gastrointestinal adverse effects within the first two years of medication. These gastrointestinal adverse effects are the most common side effects associated with MTX therapy [25]. In a 2014 report, it was revealed that gastrointestinal complaints, such as vomiting, nausea, diarrhea, anorexia, and abdominal distress, were observed frequently [78]. In addition, a study discovered a higher incidence of diarrhea in RA patients who receive oral MTX medication [79]. Some recent investigations, such as a study in 2016, have revealed that MTX usage can cause mucocutaneous ulcers in the mouth, specifically in a number of individuals who also tested positive for the Epstein-Barr virus [62]. The pathogenic pathway that causes gastrointestinal side effects involves several organs. There is a relationship between the appearance of adverse effects and variations in plasma homocysteine [80,81]. Treatment of MTX poisoning, when caused by a decrease in the folate level, based on individual symptoms, such as nausea and vomiting, can be avoided by adding folic acid to MTX medication. Folinic acid is used to decrease the impact of MTX toxicity on the gastrointestinal tract. Individuals with renal failure who have been poisoned with MTX may be cured with charcoal or cholestyramine [82,83].

Management of MTX toxicity

Patients who exhibit potential MTX poisoning symptoms and signs should be hospitalized in an intensive care environment, and treatment professionals should monitor reverse barrier nursing. Management of severe MTX toxicity has three main goals: clearance of MTX from the bloodstream, folinic acid therapy, and organ treatment [84].

Folinic Acid Rescue

The preferred antidote for MTX poisoning is folinic acid. This treatment plan reduces the toxicity of MTX while replenishing intracellular folate stocks. The ability to measure blood MTX levels is not generally accessible, and most cases are handled using a diagnosis-based approach; this is despite the fact that serum MTX concentrations should be assessed in all acute cases of MTX poisoning. A folinic acid recovery treatment plan needs to be guided by serum MTX concentrations. MTX levels should be taken into account when adjusting the dose of folinic acid. Until levels drop below 0.2 μmol/L, serum MTX concentrations should be checked every 24 hours. Furthermore, the monitoring of MTX medication is not necessary in the case of low-dose poisoning caused by weekly doses of 5 to 25 mg of MTX [85].

Hydration

The kidneys remove the highest amount of MTX (over 90%). To maintain urine production and to aid in the elimination of MTX, a suitable diuresis of 600 mL of urine over six hours or 200 mL of urine over two hours must be maintained. Fluid equilibrium should be closely managed to avoid nephrotoxicity and excess fluid. The aim is to achieve a urine output of around 2 L/m^2^ per day until MTX levels drop to 0.2 μmol/L [86].

Urine Alkalization

At acidic pH values, MTX and its metabolites (2,4-diamino-N10-methylpteroic acid (DAMPA)) are weakly soluble. Increased urinary pH by 6.0 to 7.0 enhances MTX and also its solubility of metabolites by about five to eight times and prevents crystal formation. The probability of the intratubular crystallization process is reduced by urinary alkalinization with 40-50 mEq sodium bicarbonate for every liter of intravenous fluid. MTX and its metabolites (7-OH-MTX) have a high dosage solubility as the pH rises from 5.0 to 7.0. The urine pH should be greater than 7.0 to facilitate the elimination of MTX and avoid the crystallization of MTX. It must be kept at this dose throughout the therapy phase and until concentrations are 0.2 μmol/L or less [84,86].

Managing Delayed MTX Excretion

Tubular re-absorption, glomerular filtration, and tubular secretion all contribute to MTX elimination in the kidneys. However, toxic amounts of MTX endanger the renal tubules, leading to impaired renal excretion. The primary mechanisms of MTX-induced renal damage include crystal nephritis and direct tubular damage [87]. Glucarpidase (carboxypeptidase G2) has been licensed for the treatment of high plasma MTX levels of >1 μmol/L in patients with compromised renal function who have delayed MTX elimination [88]. If blood MTX concentrations are more than 10 μmol/L and creatinine increases by 100% or more from the last dose of MTX, glucarpidase should be explored. Glucarpidase can decrease blood MTX levels by 97% or more within 15 minutes. Furthermore, glucarpidase therapy has little effect on intracellular MTX levels, so high-dose folinic acid must be administered as well [87,88]. The cost of glucarpidase is so exorbitant ($42,000.00/1,000 units) that even at big hospitals, accessibility and availability are difficult since the medicine is used seldom and is not maintained "in stock." Usually, pharmacies require at least 24 hours' notice to get the agent.

Leucovorin Rescue

Leucovorin has been considered the basis for HD-MTX therapy for more than 30 years. Leucovorin is highly beneficial in preventing myelosuppression, gastric toxicity, and neurotoxic effects after HD-MTX therapy. HD-MTX-containing chemotherapy procedures also include guidelines for the timing and dose of leucovorin treatment to prevent cell damage [86]. Because leucovorin efficiently neutralizes methotrexate effects, it should not be started too soon because it will reduce not only toxicity but also anticancer effectiveness. In this context, if a person receives leucovorin just before the time HD-MTX therapy is scheduled to begin, leucovorin must be stopped, and HD-MTX must be delayed until the following day [11,86].

Other Supportive Care Measures

Additional forms of supportive treatment can be customized to specific patient health conditions. For example, in individuals with pre-existing kidney failure or severe poisoning from a previous course of HD-MTX, dosages can be reduced, and serum MTX concentrations can be monitored early to ensure that there is no excess buildup [12]. Patients taking HD-MTX must also avoid other nephrotoxins [12,89].

MTX dosing in renal deficiency

A lower initial dose is recommended for patients with an estimated glomerular filtration rate (eGFR) of 30 to 59 mL/minute, and a more gradual dose increase, close monitoring, and a lower maximum weekly dose should be taken into account, depending on the overall clinical status. MTX is contraindicated in RA patients if the eGFR is less than 30 mL/minute [90]. Although MTX in low doses is not nephrotoxic, it is almost eliminated by the kidneys. As a result, it is prudent to keep an eye on renal function and adjust the dosage or discontinue the medication if renal insufficiency manifests. The maximum dose is determined by the clinical response of the patients, as well as by their age, comorbidities, and the severity of disease activity [90,91]. According to Kintzel and Dorr, MTX dosage change is unnecessary if patient creatinine clearance (CrCl) is greater than 60 mL/minute. If CrCl is 46 to 60 mL/minute, 65% of the regular dose is used. If CrCl is 31 to 45 mL/minute, half the regular dose is provided; if CrCl is <30 mL/minute, usage is avoided [92].

## Conclusions

Although MTX is effective in treating many diseases, such as RA and psoriasis, and many types of malignant tumors, its misuse, whether intentional or erroneous, especially in the elderly and children, may cause many serious side effects, such as pulmonary toxicity, nephrotoxicity, hepatotoxicity, hematological toxicity, and an increased risk of infections. Management of severe MTX toxicity has three main goals: clearance of MTX from the bloodstream, folinic acid therapy, and organ treatment. Leucovorin is highly beneficial in preventing myelosuppression, gastric toxicity, and neurotoxic effects after HD-MTX therapy. The preferred antidote for MTX poisoning is folinic acid. Glucarpidase has been licensed for the treatment of high plasma MTX levels of >1 μmol/L in patients with compromised renal function who have delayed MTX elimination. Patients should be aware when using MTX, and physicians should emphasize MTX when writing prescriptions to alert patients and should write instructions on prescriptions about its use to avoid medication errors resulting in MTX toxicity.
